# Education and Training in Clinical Psychology and Psychological Psychotherapy in Switzerland

**DOI:** 10.32872/cpe.v2i3.2991

**Published:** 2020-09-30

**Authors:** Marius Rubo, Chantal Martin-Soelch, Simone Munsch

**Affiliations:** a Clinical Psychology and Psychotherapy, Department of Psychology, University of Fribourg, Fribourg, Switzerland; b Clinical Psychology and Health Psychology, Department of Psychology, University of Fribourg, Fribourg, Switzerland; University of Vienna, Vienna, Austria

**Keywords:** education in clinical psychology, psychotherapy training, Switzerland, employment models, reimbursement, ordering model

## Abstract

Switzerland offers Education in Clinical Psychology in the German and French language and training in Psychotherapy in German, French and Italian. Both education and training are structured along centralized guidelines and recognized at a federal level. After finishing one’s studies, becoming a Psychological Psychotherapist requires between two and six years of postgraduate training and a financial investment of tens of thousands of Swiss Francs. Historically, it is quite common for Swiss psychotherapy trainings to incorporate a mix or combination of several psychotherapy schools such as cognitive behavioral, psychodynamic, systemic and humanistic. Foreign degrees obtained in EU countries are generally recognized, and the fulfillment of criteria is evaluated on an individual basis. Graduates find a diverse job market with opportunities to work in clinics and psychotherapeutical practices, but the absence of direct reimbursement via mandatory health insurance plans for psychological psychotherapists (not psychiatrists) lead many to work on patients’ private payments or as a psychiatrist’s employee. The ordering model, a potential new regulation allowing for the direct reimbursement of psychological psychotherapists’ work, is planned to be decided upon throughout 2020.

## Abstract (highlights)

- Switzerland offers education in Clinical Psychology in German and French.
- Trainings in Psychological Psychotherapy often incorporate content from various Psychotherapy schools.
- Degrees obtained in EU countries can be acknowledged.
- Several employment models exist for Psychological Psychotherapists, and the profession hopes to see improvements in the reimbursement situation throughout this year.

## Education in Clinical Psychology

### Goals

In Switzerland – a federal parliamentary republic consisting of four broad geographic and language regions and of 26 cantons – private and state universities as well as universities of applied sciences are centrally evaluated by the governmental institution swissuniversities (www.swissuniversities.ch). Altogether 12 universities are currently accredited and fulfill the criteria of the federal higher education law (Hochschulförderungs- und Koordinationsgesetz, HFKG) and six of them (Universities of Basel, Bern, Fribourg, Geneva, Lausanne and Zurich, but also the Zurich University of Applied Sciences) offer education programs in Clinical Psychology in German or French language. The University in Fribourg furthermore offers a bilingual curriculum (German-French) with courses in English.

Similar to most countries in Europe, the Swiss education in Clinical Psychology includes a three years’ Bachelor and a two years’ Master program. The Bachelor program includes basics of psychology such as human cognition, experimental psychology, personality, development, emotions, and psychopathology. Subsequent Master programs in Clinical Psychology focus on psychopathological and related biological processes, knowledge on evidence-based diagnostic and interventions and more strongly emphasize the ability to critically assess and process the scientific literature in the field. These skills allow students to pursue careers both in clinical settings (and particularly to pursue a federally accredited postgraduate training in psychotherapy or health psychology) as well as in research. A master diploma in psychology leads to the title “psychologist” that is recognized at federal level.

### Contents, Structure and Costs

The contents of Bachelor programs in Psychology and Master programs in Clinical Psychology are similarly structured and comparable across all Swiss universities according to the guidelines of the *Konferenz der Schweizer Psychologie-Institute (K-PSYCH)*, which will be updated in June 2020. Bachelor programs include three years of studies and 180 ECTS, while master programs consist of two years of studies and 120 ECTS. A two-month full time practical experience which is mandatory in master programs can be completed in clinical settings, but also in research groups.

### Evaluation

The Bachelor program in Psychology includes three consecutive years of studies. After the first year, students are required to pass written propaedeutic exams (except at the FernUni, ZHAW and FHNW). Subsequent examinations during the second and third year are individually organized by the universities and include oral and written exams as well as written essays or presentations.

### Costs

Swiss Universities open all their education programs for a semester fee from CHF 500 up to CHF 1300 (swissuniversities, n.d.). Granting of studentships depends on the parental income and eligibility is usually organized by the canton of domicile of the student.

## Legal Framework

### Swiss Education in Psychology, Clinical Psychology and Psychotherapy

In 2013, the law on Psychology Professions (Bundesamt für Gesundheit [BAG], 2020a, PsyG/LPsy) was introduced with the overall purpose of reinforcing public health and protecting customers and people in need for psychological opinion, counseling or treatment from fraud. With the new law, the title “Psychologist” is now protected in Switzerland. Obtaining a master degree in Clinical Psychology in Switzerland qualifies students to enter accredited postgraduate specialized trainings in Neuropsychology, Psychological Psychotherapy, Health psychology, Clinical Psychology and Children and Youth Psychology. These are the 5 specialized post-graduate titles defined in the PsyG/LPsy. All Swiss postgraduate trainings are evaluated and accredited by the federal Commission on Psychology Professions (BAG, 2019b). This Commission also evaluates and decides the recognition of foreign degrees. Following the implementation of the PsyG in 2013 and until end of 2018, all existing training programs in Psychological Psychotherapy from different stakeholders in Switzerland underwent an evaluation process, which is required to be repeated every seven years, under the lead of the Commission of Psychology Professions.

### Recognition of Foreign Degrees

Relying on the Swiss-EU Bilateral Agreement on the Free Movement of Persons (AFMP), Switzerland has adopted the EU’s system of mutual recognition of professional qualifications (State Secretariat for Education, Resarch and Innovation [SERI], n.d.), in which a university degree or a degree from a university of applied science from abroad is recognized if it is acknowledged in the country of origin. Nonetheless, each application is evaluated on an individual basis and additional requirements may be determined before a title is validated as equivalent. Requests from countries outside of Europe are processed equally. As Switzerland is relatively unique in Europe in requiring 5 years of advanced training, additional parts of training regularly have to be caught up here.

### Register of Psychology Professions

Psychologists with a title in Psychological Psychotherapy (and any other postgraduate training accredited by the Federal Department of Health as e.g. Child and Youth Psychologist, Neuropsychologist, Health Psychologist and Clinical Psychologists) are obliged to enlist in the Register of Psychology Profession (BAG, 2020b). In the case of Psychological Psychotherapists, the list includes information about whether the person is entitled to autonomously offer psychotherapeutic treatment. The register aims at increasing the transparency of offers across cantons and to ensure the quality of treatment offers to the Swiss inhabitants. The completion of the register is currently still ongoing.

## Training in Psychological Psychotherapy

### Diverse Options for Therapy Trainings

In Switzerland, different institutions offer training programs in Psychological Psychotherapy. In 2013, after the introduction of the PsyG/LPsy, a total of 62 postgraduate curricula in Psychological Psychotherapy were accredited temporarily until 2018, which means that these diplomas were recognized by the government independent of an evaluation according to the before mentioned conditions. These psychotherapy training offers were diverse and encompassed cognitive-behavioral, humanistic, psychodynamic and systemic approaches. Until 2019, 41 of these initial programs have been accredited by the Federal Department of Health. Currently, three German universities, two French universities, one German/French university and the ZHAW University of Applied Sciences from Switzerland offer a total of 12 postgraduate psychotherapy trainings for applicants holding an accredited Master's degree. The remaining 29 postgraduate psychotherapy trainings are offered outside the university. Of the 41 psychotherapy trainings, the following therapy schools are represented: (1) 8 in cognitive behavioral therapy; (2) 11 in psychoanalytic therapy; (3) 10 in systemic therapy; (4) 4 in humanistic methods; (5) 8 in various mixed forms and integrative approaches. Notably, it is common for the abovementioned programs to incorporate content from other “schools”.

In Switzerland, adult and child/adolescent psychotherapy are currently not considered to be separate psychological professions by law. Therefore, a postgraduate diploma in Psychological Psychotherapy entitles psychotherapists to offer treatment to the full age range. Nevertheless, some training programs focus more on adults whereas others focus explicitly on children, adolescents and young adults.

### Goals

Postgraduate trainees are expected to have established a profound understanding of human experience and behavior as well as their biological underpinnings during the Bachelor and Master program in Clinical Psychology. They are already skilled to assess and evaluate complex human experience and behavior in diverse developmental stages and psychosocial contexts. Building on these skills, postgraduate trainings in Psychological Psychotherapy (PPT) then teach to autonomously offer and evaluate psychotherapeutic treatment. Specifically, trainees learn to employ evidence-based psychotherapeutic theories, techniques and methods, reflect professional activities based on theoretical and practical expertise and reflecting societal and legal aspects, cooperate with other health experts, respect cost-efficiency in their professional activities, and others.

### Contents and Structure

Altogether, obtaining the title of a Psychological Psychotherapist requires between four and six years of fulltime postgraduate training and is prolonged if the training is executed in part time. Resulting in a sum of 5430 units (one unit equals 45 minutes), the training consists of theory and competences (500 units), Supervision (at least 150 units, 50 of which in a single setting), self-experience (at least 100 units, 50 of which in a single setting), individual and practical experiences under supervision (at least 500 units, with at least 10 case reports), and altogether at least 2 years of fulltime practical experiences in an institution of primary psychosocial health care, with at least one year in psychotherapeutic or psychiatric primary health care (EDI, 2016). In case of part time employment, the duration is automatically prolonged. No less than 50% part time employment is allowed. These are basic and mandatory requirements. Most institutions offering psychotherapeutic training ask for more hours than legally required, especially for more units of theory and practical competence training.

### Evaluation

At the end of postgraduate trainings in Psychological Psychotherapy, the responsible teaching and supervising experts of the program examine the trainees’ theoretical and clinical competences and evaluate whether all units have been acquired. During the training process, supervisors and experts repeatedly comment on the trainee’s professional development and their patients’ therapeutic processes and discuss the trainees’ case reports Examination procedures during or at the end of the program depend on the individual institute offering the postgraduate training and may include oral or written theoretical exams and oral exams on case reports of the trainees.

### Costs

The total costs of postgraduate psychotherapy training vary strongly, ranging from a minimum of 35200 CHF to a maximum of 91700 CHF.

### Number of Psychologists and Psychotherapists

According to the most recent representative survey initiated by the Swiss Federation of Psychologists (FSP, https://www.psychologie.ch; Stettler, Stocker, Gardiol, Bischof, & Künzi, 2013) in 2012, Switzerland counted 15 000 psychologists or 1.8 psychologists per 1000 inhabitants, while there were 0.4 fulltime working Psychological Psychotherapists per 1000 inhabitants. In 2012, 32% of all Psychological Psychotherapists reported working according to the psychoanalytic, 19% to the cognitive-behavioral, 17% to the humanistic and 12% to the systemic orientation, and an additional fraction reported to adhere to multiple schools (Grosse Holtforth, Kramer, & Dauwalder, 2015). In 2019, around 8600 (79% female) students were enrolled in Psychology at a Swiss university (not including PhD students and persons pursuing postgraduate trainings (Bundesamt für Statistik, 2019).

After the implementation of the law on Psychology Professions, from April 2013 until December 2019, a total of 2218 degrees in psychology and 359 degrees in psychotherapy from abroad have been accredited. Altogether 80% of these candidates had pursued their education and psychotherapy training in Italy, Germany, France, Portugal and in Austria. The remaining 20% applications came from South America and from Mid and Eastern Europe (BAG, 2020c).

### Advanced Training for Psychotherapists

After receiving a diploma in Psychological Psychotherapy from an accredited training program, psychotherapists are obliged to participate in regular advanced trainings in order to refresh and renew their theoretical and practical competences. Nevertheless, up to date, neither contents nor hours of advanced training have been defined.

## Trainings in Other Specialization Titles

For Neuropsychology, there is one accredited curriculum in French and one accredited training in German, offered in collaboration between Universities and the Swiss Society of Neuropsychology. Neuropsychologist are the only specialized psychologists reimbursed by the mandatory health insurance.

For Health Psychology, there is at the moment only one French-speaking curriculum offered by French-speaking Universities (Fribourg, Geneva and Lausanne, Leading House Fribourg) in collaboration with the Swiss Society for Health Psychology under the institutional cover of the rector conference of French-speaking Universities in Switzerland (the so-called Triangle Azur). The delivered title is a MAS in Health Psychology, the accreditation process will begin soon.

For Clinical Psychology, there is one French-speaking curriculum offered by 3 French-speaking Universities (Geneva, Lausanne and Fribourg, Leading House Geneva) in collaboration with the Swiss Association of clinical psychologists, also under the institutional cover of the rector conference of French-speaking Universities in Switzerland (the so-called Triangle Azur). The delivered title is a MAS in Clinical Psychology, the accreditation process will begin soon. The Swiss Association of clinical psychologists offer a complete curriculum in German and one in Italian, leading the title of specialist in clinical psychology recognized by the Swiss Federation of Psychologists, but not accredited by the federal Commission on Psychology Professions yet. The Clinical Psychology specialization is particularly aimed at the employment in mental health hospitals with in-patients. For Children and Youth Psychology, there is only one training option offered by the Swiss Association of Children and Youth Psychologists, that is not accredited yet.

## Employment Situation for Psychological Psychologists in Switzerland

### Psychotherapy in the Swiss Health System

The total annual costs for health services which are reimbursed by mandatory insurances in Switzerland amount to CHF 9,86 billion of which 11% (CHF 1,08 billion) are generated in the field of psychiatry (including Psychological Psychotherapy). Specifically, 2.9% of the total annual health costs covered by mandatory insurances (CHF 286 million) are generated by psychological services (Grosse Holtforth, Kramer, & Dauwalder, 2015). These numbers do not include the costs of Psychological Psychotherapy covered by private insurances or paid by patients personally.

According to a study by the *Schweizerisches Gesundheitsobservatorium*, around 470,000 individuals (7% of the population above 15 years of age) sought psychotherapeutic treatment in 2009, 88% of who were treated in an outpatient and 12% in an inpatient setting (Rüesch, Baenziger, & Juvalta, 2013). Psychological Psychotherapists in particular, treat 259 000 of these patients each year (Stettler et al., 2013). On average, each Psychological Psychotherapist treats 84 patients per year, and each patient receives 29 sessions within 17 months of treatment. A majority of Psychological Psychotherapists reports having a wait list (59%). 43% therapists report that they do not have any current availability (Stettler et al., 2013). Quantitative research on treatment gaps in psychotherapy in Switzerland is relatively scarce. Estimates of the percentage of individuals suffering from a mental disorder who do not receive even minimal treatment range from 40% to 65% (Stocker et al., 2016).

The reimbursement of Psychological Psychotherapy in the Swiss health-care system is divided into three main financing sources. Firstly and most importantly, 67% of the psychotherapeutic services are reimbursed by the mandatory health insurance plans. Secondly, 29% of the psychotherapeutic services are paid by the patients themselves or by their private complementary insurances, and, thirdly, 4% of the psychotherapeutic services are financed by public social services. (Stettler et al., 2013).

### Employment Models for Psychological Psychotherapists

The fact that there is so far no direct reimbursement of Psychological Psychotherapy by the mandatory health insurances influences the current employment models. About a third of all Psychological Psychotherapists work in private practice, where their patients privately pay for psychotherapeutic treatment or receive partial reimbursement via a private insurance plan. Another group of Psychological Psychotherapists of about 40% work in so-called “delegated” practice (Stettler et al., 2013). As a “delegated psychotherapist” the Psychological Psychotherapist is an employee of and works in the rooms of a psychiatrist. According to the current legislation, this means that the psychiatrist “delegates” psychotherapy and that the Psychological Psychotherapist works under the psychiatrist’s legal responsibility and supervision. The psychiatrist gets reimbursed for the psychotherapy provided by the psychologist via mandatory basic insurance plans. The payment of the Psychological Psychotherapists varies from employer to employer (psychiatrist). Strikingly, the reimbursement for delegated psychotherapy is only around two thirds of that for psychotherapy offered by psychiatrists.

Psychological Psychotherapists further work in outpatient clinics within larger institutions, where patients either pay privately (if a psychological psychologist is head of the unit), or the patients get reimbursed for their psychotherapies (if a psychiatrist is head of the clinic). Finally, around 13% of psychological psychotherapists work in psychiatric hospitals and provide primary mental health care as well as psychotherapy.

### (Possibly) Better Working Conditions in the Future

The system of delegated psychotherapy is highly controversial in Switzerland. It was originally implemented as a temporary solution to improve access to mental health care until the psychological profession were regulated in detail and – again supposedly temporarily - treats psychological psychotherapists as auxiliary employees of psychiatrist-psychotherapists receiving a lesser payment. Although the rationale behind the delegated model has become obsolete with the „Law on Psychology Professions“ introduced in 2013, adaption has been postponed until today. Remarkably, the delegation model has remained unchanged despite clearly standing at contrast with psychological psychotherapists’ official authorization to execute their profession independently and to their own full responsibility (www.psyeg.admin.ch). The Law however changed the situation of Neuropsychologists who are now reimbursed by the mandatory Law.

Recently, a potential new regulation called the “ordering model” is being discussed and evaluated by the government. In the ordering model, a psychological psychotherapist would work self-employed and in his/her own office, and a physician’s prescription would suffice for the reimbursement of a limited number of psychotherapy sessions by mandatory health insurance plans. After a period of more than 7 years of internal evaluations, the federal council opened a consultation phase regarding the planned new legal regulations in July 2019 (BAG, 2019a).

While the federal department of health supports the new regulation, there have been heated debates since the consultation phase has been opened, most prominently between psychologists, psychiatrists and politics. As a result, the decision on the implementation of the ordering model, which was originally scheduled for early 2020, has been postponed and seems unlikely to be processed in due time.
